# A Markov Chain Monte Carlo Approach to Estimate AIDS after HIV Infection

**DOI:** 10.1371/journal.pone.0131950

**Published:** 2015-07-06

**Authors:** Ofosuhene O. Apenteng, Noor Azina Ismail

**Affiliations:** Department of Applied Statistics, Faculty of Economics & Administration, University of Malaya, Kuala Lumpur, Malaysia; University of Athens, Medical School, GREECE

## Abstract

The spread of human immunodeficiency virus (HIV) infection and the resulting acquired immune deficiency syndrome (AIDS) is a major health concern in many parts of the world, and mathematical models are commonly applied to understand the spread of the HIV epidemic. To understand the spread of HIV and AIDS cases and their parameters in a given population, it is necessary to develop a theoretical framework that takes into account realistic factors. The current study used this framework to assess the interaction between individuals who developed AIDS after HIV infection and individuals who did not develop AIDS after HIV infection (pre-AIDS). We first investigated how probabilistic parameters affect the model in terms of the HIV and AIDS population over a period of time. We observed that there is a critical threshold parameter, *R*_0_, which determines the behavior of the model. If *R*_0_ ≤ 1, there is a unique disease-free equilibrium; if *R*_0_ < 1, the disease dies out; and if *R*_0_ > 1, the disease-free equilibrium is unstable. We also show how a Markov chain Monte Carlo (MCMC) approach could be used as a supplement to forecast the numbers of reported HIV and AIDS cases. An approach using a Monte Carlo analysis is illustrated to understand the impact of model-based predictions in light of uncertain parameters on the spread of HIV. Finally, to examine this framework and demonstrate how it works, a case study was performed of reported HIV and AIDS cases from an annual data set in Malaysia, and then we compared how these approaches complement each other. We conclude that HIV disease in Malaysia shows epidemic behavior, especially in the context of understanding and predicting emerging cases of HIV and AIDS.

## Introduction

Acquired immune deficiency syndrome (AIDS), caused by infection with human immunodeficiency virus (HIV), is one of the most alarming and deadly diseases in human history. The total number of people living with HIV and AIDS in 2013 was 35 million [1]. The spread of AIDS through populations has caused panic and economic disturbance. In the last three decades since the emergence of AIDS, many interdisciplinary scientific efforts have coalesced to model the spread of this disease. In 1989, Hyman and Stanley [2] generated mathematical models based on the underlying transmission mechanisms of HIV/AIDS that were used to understand and anticipate its spread in different populations. In 1991, Romieu et al. [3] presented work demonstrating how to model the spread of HIV/AIDS in Mexico City; the goal of their work was to provide a conceptual framework to help understand the transmission dynamics of infection and give a reasonable estimation of the short-term cumulative number of AIDS cases. Mathematical modeling of the spread of HIV/AIDS has become even more useful in the modern era of AIDS research. In 2011, Nyabadza [4] presented a simple deterministic HIV/AIDS model that applied ordinary differential equations to the current South African situation and considered the use of condoms, sexual partner acquisition, behavior change, and treatment; their results suggested that HIV/AIDS could be controlled through these measures. Naresh [5] calculated the spread of the AIDS epidemic with immigration among HIV-infected individuals, and the findings revealed a constant flow of immigrating susceptible individuals and individuals infected with HIV. Merli [6] presented an exploration of the implications of patterns of sexual behavior for the spread of HIV in China; this model reflected the uncertainty surrounding key parameters, and the analyses used showed a range of possible outcomes. In 1999, Kakehashi formulated a mathematical model to describe the spread of HIV/AIDS among adult commercial sex workers in Japan that was used to analyze the effect of introducing HIV-infected commercial sex workers into a population without HIV [7]. De Arazoza and Lounes (2002) outlined how a non-linear model could be used to develop an epidemic with contact tracing, specifically in Cuba. These authors suggested that to control the spread of HIV/AIDS, the target group must be in contact with individuals who carry HIV [8]. In 2008, Kim [9] formulated a simple continuous model for the transmission of HIV, although this model failed to consider the demographic parameters that have a significant impact on modeling the spread of HIV. Furthermore, most of these previous models have serious drawbacks. For instance, most of these models failed to demonstrate how the impact of AIDS causes the death of HIV-infected individuals. These models also typically describe changes in time and are therefore referred to as ‘dynamic’ models, where time is the independent variable. Similar work was conducted by Haario et al. (2006) [10], who proposed various strategies to combine two quite powerful ideas in the Markov chain Monte Carlo method (MCMC), adaptive Metropolis samplers and delayed rejection, to study the spread of algae.

The current study assessed the robustness of a new method for predicting the spread of AIDS among HIV-infected individuals. We used Monte Carlo-based methods, including importance sampling and MCMC approaches, which are more useful in dealing with the nonlinearity and interdependency of parameters through their application to a model describing the dynamics of HIV [11]. MCMC is one of the most important numerical techniques for creating a sample from the posterior distribution, and it has been widely used in mathematical modeling to quantify parameter uncertainties [10,12,13]. In the current study, we formulated a deterministic mathematical model to reflect the trend of AIDS cases after HIV infection, and we also applied MCMC approaches by considering the uncertainty in the model parameters and the model output to supplement the mathematical model.

## Materials and Methods

**Fig 1:**
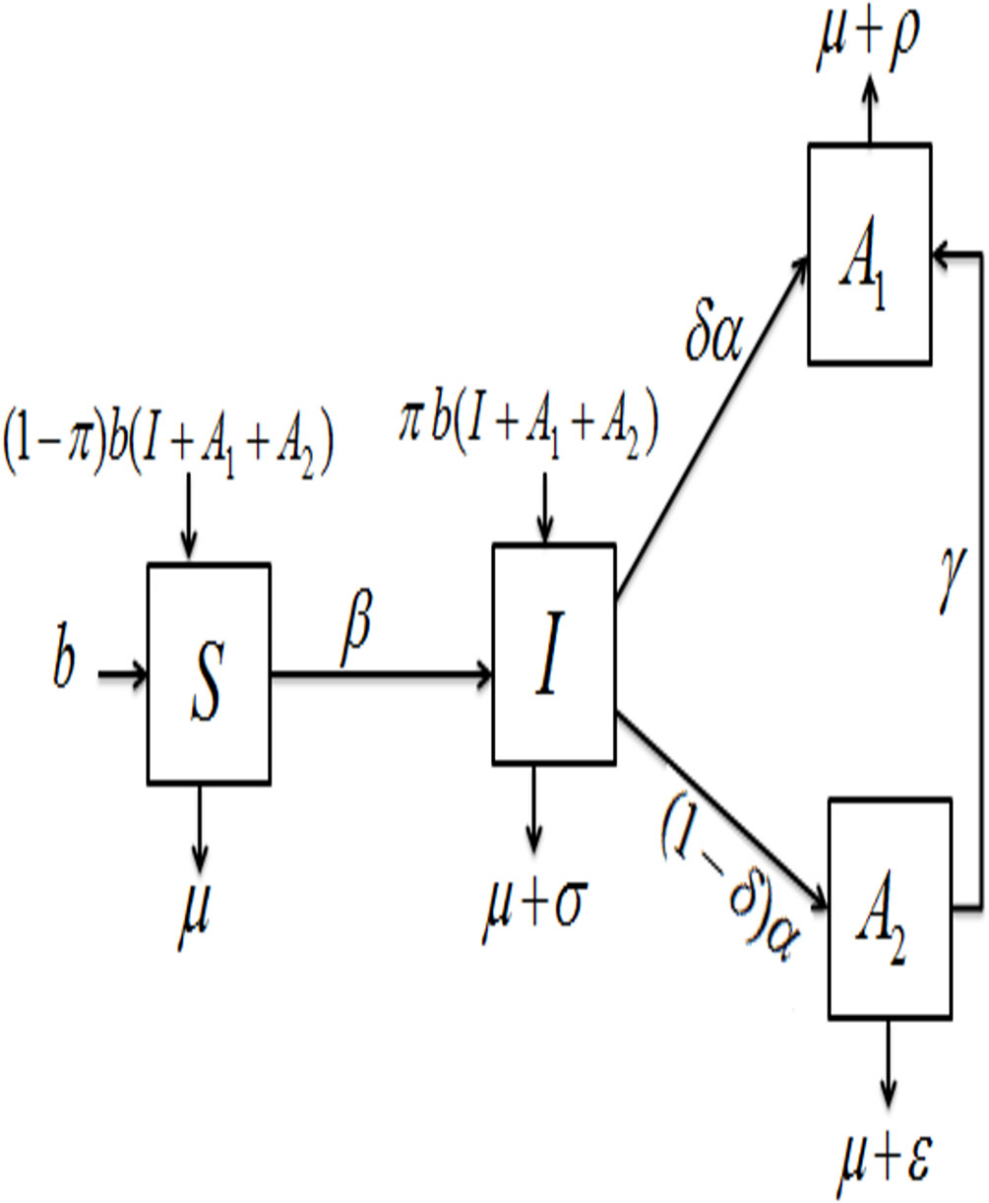
Schematic representation of the *SIA*_1_ *A*_2_ model. The flow chart of the *SIA*_1_ *A*_2_ model.

We present the simplest HIV disease models where individuals classified as a sexually active population are divided into four classes: susceptible, *S*(*t*); infected, (HIV) *I*(*t*); pre-AIDS cases who did not progress to AIDS after HIV infection, *A*_1_(*t*); and AIDS cases who have AIDS after HIV infection at time *t*, *A*_2_(*t*). HIV can be transmitted to a susceptible person when they come into contact with an infected person via the appropriate transmission routes. In 2003, Rao [14] formulated a model for individuals who did or did not develop AIDS after the HIV epidemic in India. Unlike the model from this report [14], our model assumed that *γ* is the rate at which an individual will fully move from *A*_1_(*t*) class to *A*_2_(*t*) class, which is a very significant indicator of when an intervention should be introduced. We assumed that the infected individuals are capable of having children that are either infected with HIV or will not have HIV. However, the susceptible class has a recruitment rate equivalent to the birth rate, *b*, which is independent of vertical transmission. Moreover, this model assumes that infected newborn babies enter the HIV class at the rate of *b*(*I* + *A*_1_ + *A*_2_), for which we assume that *I*, *A*_1_, and *A*_2_ are sexually active, and *πb*(*I* + *A*_1_ + *A*_2_) are individuals who are infected and enter the HIV stage. The portion *π* of these individuals is assumed to be infected with HIV (categorized in the *I* class), and the complementary portion (1 − *π*)*b*(*I* + *A*_1_ + *A*_2_) is considered susceptible (and moves to the susceptible class *S*). The removal rate of infected HIV individuals who enter the AIDS class is represented by *α*; the portion of HIV-infected individuals is *δ*. This model also assumes that at rate *δα*, some of the HIV-infected cases transition to the AIDS group, whereas the remaining HIV-infected cases move to the class of individuals who do not develop AIDS (pre-AIDS) after an HIV infection rate of (1 − *δ*)*α*, where 0 ≤ *δ* ≤ 1. The model also assumes the natural death rate *μ* of individual deaths from all four compartments. *β* is the contact rate between susceptible individuals and exposed or HIV-infected individuals. AIDS patients are given an additional disease-induced mortality rate: *σ* > 0, *ε* > 0 and *ρ* > 0 for *I*(*t*), *A*_1_(*t*) and *A*_2_(*t*), respectively. This form of a susceptible–infected–pre-AIDS–AIDS (*SIA*_1_ *A*_2_) model can be used to model HIV disease based upon the assumption that once an individual becomes infected, that individual remains infectious for life, as shown in Fig 1.

The deterministic systems of nonlinear differential equations describing the *SIA*_1_ *A*_2_ models of HIV disease with additional demographics (birth and death) for an individual population are listed as follows:

$$\frac{dS}{dt}=bS+(1-\pi )b(I+A_{1}+A_{2})-\beta IS-\mu S$$ (1)

$$\frac{dI}{dt}=\pi b(I+A_{1}+A_{2})+\beta IS-(\mu +\alpha +\sigma )I$$ (2)

$$\frac{dA_{1}}{dt}=(1-\delta )\alpha I-(\mu +\varepsilon +\gamma )A_{1}$$ (3)

$$\frac{dA_{2}}{dt}=\alpha \delta \;I-(\mu +\rho )A_{2}\;+\gamma {\text{A}}_{1}$$ (4)

The summation of 1)–(4) and substituting *S* = *N* − *I* − *A*_1_ − *A*_2_ into (2) are given by

$$\frac{dN}{dt}=bN-\mu N+(\alpha -\sigma )I-\varepsilon A_{1}-\rho A_{2}$$ (5)

$$\frac{dI}{dt}=\pi b(I+A_{1}+A_{2})+\beta IS-(\mu +\alpha +\sigma )I$$ (6)

$$\frac{dA_{1}}{dt}=(1-\delta )\alpha I-(\mu +\varepsilon +\gamma )A_{1}$$ (7)

$$\frac{dA_{2}}{dt}=\alpha \delta \;I-(\mu +\rho )A_{2}\;+\gamma {\text{A}}_{1}$$ (8)

where *N*(*t*) represents the total population.

### MCMC approach

The MCMC method consists of algorithms for inverse modeling, in particular identifiability [15], which includes the local and global sensitivity analysis, and this was employed to estimate parameter uncertainties [10,16,17]. One of the MCMC methods suited for use with dynamic models is delayed rejection adaptive metropolis [10], which was applied to analyze how the model fits the reported HIV and AIDS cases. The MCMC method was applied to sample from the probability distribution by creating a Markov chain with a set of parameters, which combine the parameter values representing the parameter distribution at the equilibrium distribution. We set the prior distribution for the parameters to *θ* and independent variables *t* (for details see [11]). Similarly, we set *y* to represent our system of non-linear Eqs (1)–(4) model (e.g., *f*(*t*, *θ*)). We also assumed that *χ* is the additive and the independent Gaussian error, with unknown variance *σ* ^2^. These terms can be defined as follows:

y=f(t,θ)+χ (9)

$$\chi \sim N(0,\sigma^{2})$$ (10)

The posterior for the parameters is estimated as [16]

$$p\left(\theta |y,\sigma^{2}\right)\propto \text{exp}\left(-0.5\;\left(\frac{SS(\theta )}{\sigma^{2}}\right)\right)•p_{pri}(\theta )$$ (11)

where *SS* is the sum of squares function *SS*(*θ*) = ∑ (*y*_*i*_ − *f*(*t*_*i*_, *θ*_*i*_))^2^ and *p*_*pri*_ (*θ*) is the prior distribution of the parameters. To obtain proper results from the MCMC method, a constrained least squares approach is necessary to provide initial estimates of *θ*_*i*_. If the non-informative prior *p*_*pri*_ (*θ*) is constant for all of the values of (*θ*), this can be ignored.

For the reciprocal of the error variance (*σ* ^−2^), a gamma distribution is used:

$$p_{pri}(\sigma^{-2})\sim \Gamma \;\left(\frac{n_{0}}{2},\frac{n_{0}}{2}S_{0}^{2}\right).$$ (12)

The reciprocal of the error variance at each MCMC step is sampled from a gamma distribution [18] as follows:

$$p\;\left(\sigma^{-2}|\left(y,\theta \right)\right)\sim \Gamma \;\left(\frac{n_{0}+n}{2},\frac{n_{0}S_{0}^{2}+SS(\theta )}{2}\right),$$ (13)

where *n*_0_ and *n* input arguments to the function and the number of observations, respectively [11].

### Equilibrium, stability analysis and material

In this section, we describe the selection of equilibrium points to determine whether the populations are constant or changing. Additionally, we determined whether the model system was stable or unstable. At equilibrium, (5)-(8) becomes $\frac{dN}{dt}=\frac{dI}{dt}=\frac{dA_{1}}{dt}=\frac{dA_{2}}{dt}=0$.

$$bN-\mu N+(\alpha -\sigma )I-\varepsilon A_{1}-\rho A_{2}=0$$ (14)

$$\pi b(I+A_{1}+A_{2})+\beta I(N-I-A_{1}-A_{2})-(\mu +\delta \alpha +\sigma )I=0$$ (15)

$$(1-\delta )\alpha I-(\mu +\varepsilon +\gamma )A_{1}=0$$ (16)

$$\alpha \delta \;I-(\mu +\rho )A_{2}\;+\gamma {\text{A}}_{1}=0$$ (17)

From (16) and (17) we get

$$A_{1}=\frac{(1-\delta )\alpha I}{\mu +\varepsilon +\gamma }$$ (18)

$$A_{2}=\frac{\alpha I(\delta \mu +\delta \varepsilon +\gamma )}{\left(\mu +\rho )(\mu +\varepsilon +\gamma \right)}$$ (19)

By submitting (18) and (19) into (14), and solving for *I*, *A*_1_, and *A*_2_, we obtain:

$$\begin{array}{l} I=\frac{\left(\mu +\rho )(\mu +\varepsilon +\gamma )N(b-\mu \right)}{\left(\mu +\rho )\left[\left(\sigma -\alpha )(\mu +\varepsilon +\gamma )+\alpha \varepsilon (1-\delta \right)\right]+\rho \alpha (\delta \mu +\delta \varepsilon +\gamma \right)}\\ A_{1}=\frac{(1-\delta )\alpha }{\left(\mu +\varepsilon +\gamma \right)}\left\{\frac{\left(\mu +\rho )(\mu +\varepsilon +\gamma )N(b-\mu \right)}{\left(\mu +\rho )\left[\left(\sigma -\alpha )(\mu +\varepsilon +\gamma )+\alpha \varepsilon (1-\delta \right)\right]+\rho \alpha (\delta \mu +\delta \varepsilon +\gamma \right)}\right\}\\ A_{2}=\frac{\alpha (\delta \mu +\delta \varepsilon +\gamma )}{\left(\mu +\rho )(\mu +\varepsilon +\gamma \right)}\left\{\frac{\left(\mu +\rho )(\mu +\varepsilon +\gamma )N(b-\mu \right)}{\left(\mu +\rho )\left[\left(\sigma -\alpha )(\mu +\varepsilon +\gamma )+\alpha \varepsilon (1-\delta \right)\right]+\rho \alpha (\delta \mu +\delta \varepsilon +\gamma \right)}\right\} \end{array}$$

### Local stability of the equilibrium point

To determine the stability of the endemic equilibrium, we assessed the eigenvalues of the characteristic equation of the corresponding Jacobian matrix, $J(N^{*},I^{*},A_{1}^{*},A_{2}^{*})=J(E^{*})$, which is given by

$$J(E^{*})=\left[\begin{array}{cccc} b-\mu & \alpha -\sigma & -\rho & -\varepsilon \\ 0 & \pi b+\beta N-(\mu +\alpha +\sigma ) & \pi b & \pi b\\ 0 & (1-\delta )\alpha & 0 & -(\mu +\varepsilon +\gamma )\\ 0 & \delta \alpha & -(\mu +\rho ) & \gamma \end{array}\right]$$ (20)

The characteristic polynomial equation corresponding to *J*(*E* ^*^) is given by

$$f(\lambda )=(\lambda_{1}-b+\mu )(\lambda_{2}+\alpha +\sigma +\mu -\pi b-\beta N)(\lambda_{3}-0)(\lambda_{4}-\gamma )=0$$ (21)

where

$$\begin{array}{l} \lambda_{1}=b-\mu ,\\ \lambda_{2}=\pi b+\beta N-(\mu +\alpha +\sigma ),\\ \lambda_{3}=0,\\ \lambda_{4}=\gamma . \end{array}$$

By substituting the appropriate parameter values into the eigenvalues from Table 1, we obtained *λ*_1_ = -0.0797, *λ*_2_ = 17.42408, *λ*_3_ = 0, and *λ*_4_ = 9.998e- 01 from the above Jacobian matrix at the point *E* ^*^ (17). Thus, *E* ^*^ is unstable because threshold parameter basic reproduction number *R*_0_ is unconditionally greater than unity. This result represents a major concern and an unsatisfactory indicator from the public health point of view because the aim is to stabilize the epidemic at the disease-free equilibrium.

**Table 1:** Summary of sensitivity values.

	Value	Scale	*L*1	*L*2	Mean	Min	Max
*β*	3.7e-05	3.7e-05	3.2e+00	5.3e-01	3.2e+00	0.0e+00	6.7122
*α*	1.8e-01	1.8e-01	4.7e-01	7.0e-02	3.8e-02	-7.4e-01	0.7532
*μ*	8.2e-01	8.2e-01	2.1e+01	3.2e+00	-2.1e+01	-3.1e+01	0.0000
*δ*	9.9e-01	9.9e-01	2.4e-01	4.9e-02	2.4e-01	0.0e+00	0.6339
*ρ*	2.1e-03	2.1e-03	2.5e-03	4.3e-04	-2.5e-03	-6.9e-03	0.0000
*ε*	4.2e-03	4.2e-03	2.7e-05	4.7e-06	-2.7e-05	-7.2e-05	0.0000
*σ*	1.0e-03	1.0e-03	6.6e-03	1.0e-03	-6.8e-03	-1.2e-02	0.0000
*π*	9.7e-01	9.7e-01	4.1e+00	6.1e-01	4.1e+00	0.0e+00	5.9892
*b*	7.4e-01	7.4e-01	2.0e+01	3.0e+00	2.0e+01	0.0e+00	28.8486
*γ*	9.9e-01	9.9e-01	1.6e-03	3.3e-04	1.6e-03	0.0e+00	0.0036

Where *β* = the contact rate between susceptible individuals and exposed or HIV-infected individuals, *α* = removal rate, *μ* = nature death rate, *δ* = the portion of HIV-infected individuals, *ρ* = disease-induced mortality rate of *A*_1_(*t*), *ε* = disease-induced mortality rate of *A*_2_(*t*), *σ* = disease-induced mortality rate of *I*(*t*), *π* = the portion of individuals infected with HIV, *b* = birth rate, *γ* = is the rate at which an individual will fully move from *A*_1_(*t*) class to *A*_2_(*t*) class.

Moreover, to determine whether the disease will continue to spread, we evaluated the stability of the disease-free equilibrium point. The reproduction number *R*_0_ is a threshold value that can be used to determine the stability of the disease-free equilibrium [19–23]. We write the right-hand side of system (5)-(8) as *F* − *V* with the following equations:

$$\begin{array}{l} F=\left(\begin{array}{c} 0\\ \beta IN\\ 0\\ 0 \end{array}\right) \end{array}$$ (22)

$$\begin{array}{l} V=\left(\begin{array}{l} \mu N-bN+(\alpha -\sigma )I+\varepsilon A_{1}+\rho A_{2}\\ -\pi b(I+A_{1}+A_{2})+\;\left(\mu +\alpha +\sigma \right)I\\ \;-(1-\delta )\alpha I+\left(\mu +\varepsilon +\gamma \right)A_{1}\\ \;-\delta \alpha I+\left(\mu +\rho \right)A_{2}-\gamma A_{1} \end{array}\right) \end{array}$$ (23)

Then, we consider the Jacobian matrices associated with *F* and *V*:

$$J_{F}=\left(\begin{array}{cccc} 0 & 0 & 0 & 0\\ 0 & \beta N & 0 & 0\\ 0 & 0 & 0 & 0\\ 0 & 0 & 0 & 0 \end{array}\right)$$ (24)

$$J_{V}=\left(\begin{array}{cccc} \mu -b & \alpha -\sigma & \rho & \varepsilon \\ 0 & \mu +\alpha +\sigma -\pi \text{b} & -\pi \text{b} & -\pi \text{b}\\ 0 & -(1-\delta )\alpha & \mu +\varepsilon +\gamma & 0\\ 0 & -\delta \alpha & -\gamma & \mu +\rho \end{array}\right)$$ (25)

The basic reproduction number of the system is obtained as the spectral radius of the matrix $J_{F}\times J_{V}^{-1}$ is

$$R_{0}=\frac{\beta \;N\left(\mu +\rho \right)\left(\mu +\varepsilon +\gamma \right)}{\left(\mu +\rho \right)\left\{\left(\mu +\varepsilon +\gamma \right)\left(\mu +\alpha +\sigma -\pi b\right)-\alpha \pi b\left(1-\delta \right)\right\}-\alpha \pi b\left(\delta \mu +\delta \varepsilon +\gamma \right)}.$$ (26)

### Source of data

The data source included the first reported cases of HIV/AIDS in Malaysia in 1986. The data used in this paper were based on information collected and collated by the Ministry of Health [24]. The formulated model was used to analyze the reported HIV and AIDS cases per year for Malaysia between 1986 and 2011. In 1985 and 1986, the total population in Malaysia was 15.8827 and 16.3294 million, respectively. The difference between the 1985 and 1986 populations is 446,700 individuals who are susceptible to HIV infection after exposure. In 1986, three individuals were infected with HIV, and these cases represented the initial infected *I*(0) compartment (HIV cases), and one individual developed AIDS after HIV infection (the *A*_2_(0) compartment). Thus, there were 446,696 individuals in the susceptible *S*(0) class, which indicates that the number of pre-AIDS cases after the HIV infection *A*_1_(0) class was 0.

## Results and Discussion

This section discusses the various results obtained using Malaysian data to analyze the accuracy of our model. Ten parameters were used to enable us to determine the inaccuracies of the model and to obtain better graphical representations.

Model fitting has become an art and requires a good understanding of the behavior of the applied model for the known parameters. The mathematical Eqs (1)–(4) are nonlinear and depend on constant parameters. The following sections will address the issue of determining parameter values that minimize a measure of badness-of-fit, usually a least square function or a weighted sum of squared residuals. This analysis will provide an estimate of the parameter uncertainty and will quantify the effects of that uncertainty on the data.

We chose parameters with the highest sensitivity values, as shown in Table 1.

Based on the summary statistics shown in Table 1, it is clear that parameter *ε* has the least effect on the output variables, whereas *b* shows the highest sensitivity value. This result shows that HIV-infected, pre-AIDS, and AIDS individuals are born at a rate of *b*, the newborn baby birth rate, which is more significant than the remaining nine parameters.

When $L1=\frac{\sum \left|S_{ij}\right|}{n}$ and $L2=\sqrt{\frac{\sum \left(S_{ij}^{2}\right)}{n}}$ are the *L*1 norm, this condition is referred to as the least absolute deviations, and the *L*2 norm is known as the least squares. The mean represents the mean of the sensitivity functions, the Min represents the minimal value of the sensitivity functions, and Max represents is the maximal value of the sensitivity functions, as shown in Table 2.

**Table 2:** Summary of the estimated parameters, standard errors, t values and p-values.

	Initial values	Estimates	Std. error	t value	Pr (> | t |)
*β*	3.699e-05	4.004e-05	1.089067e-05	3.56205518	9.315890e-04
*α*	1.773e-01	3.820e-01	4.294214e-02	3.45664816	1.264768e-03
*μ*	8.226e-01	9.264e-01	1.109851e-02	74.52726655	2.961994e-46
*δ*	9.861e-01	7.447e-03	2.056357e-03	3.01488412	4.348000e-03
*ρ*	2.114e-03	4.407e-06	2.366662e-01	2.54282300	7.284635e-01
*ε*	4.245e-03	6.659e-07	1.268450e+00	8.33906336	1.891227e-10
*σ*	9.973e-04	4.210e-06	2.549067e-01	18.8500850	9.762707e-01
*π*	9.890e-01	9.999e-01	7.012398e-01	1.37962601	1.750032e-01
*b*	7.369e-01	8.467e-01	5.762974e-03	127.09270183	6.022192e-56
*γ*	9.896e-01	9.998e-01	8.012397e-01	1.36972601	1.741032e-01

Where *β* = the contact rate between susceptible individuals and exposed or HIV-infected individuals, *α* = removal rate, *μ* = nature death rate, *δ* = the portion of HIV-infected individuals, *ρ* = disease-induced mortality rate of *A*_1_(*t*), *ε* = disease-induced mortality rate of *A*_2_(*t*), *σ* = disease-induced mortality rate of *I*(*t*), *π* = the portion of individuals infected with HIV, *b* = birth rate, *γ* = is the rate at which an individual will fully move from *A*_1_(*t*) class to *A*_2_(*t*) class.

Table 2 shows that all ten parameters have a small standard error, which provides a good representative of the real data of the entire population. Because the standard error is also inversely proportional to the sample size, this implies that the larger the sample size, the smaller the standard error. There were 25 data points with a degree of freedom of 23. All of the parameters have p-values less than 0.05, which suggests that the differences of overall parameters are statistically noteworthy. Because the t-values are significantly greater and because p-values are smaller as t-values get larger, there is a difference between the four compartments (i.e., *S*(*t*), *I*(*t*), *A*_1_(*t*), and *A*_2_(*t*)).

The empirical mean and standard deviation for each variable, standard error of the mean, and their respective 95% confident intervals are reported in Table 2. The disease-induced mortality rate *ε* for AIDS *A*_1_(*t*) cases that did not progress to AIDS after HIV infection at time *t* showed the highest standard deviation, which has a significant impact on the spread of AIDS.

**Fig 2:**
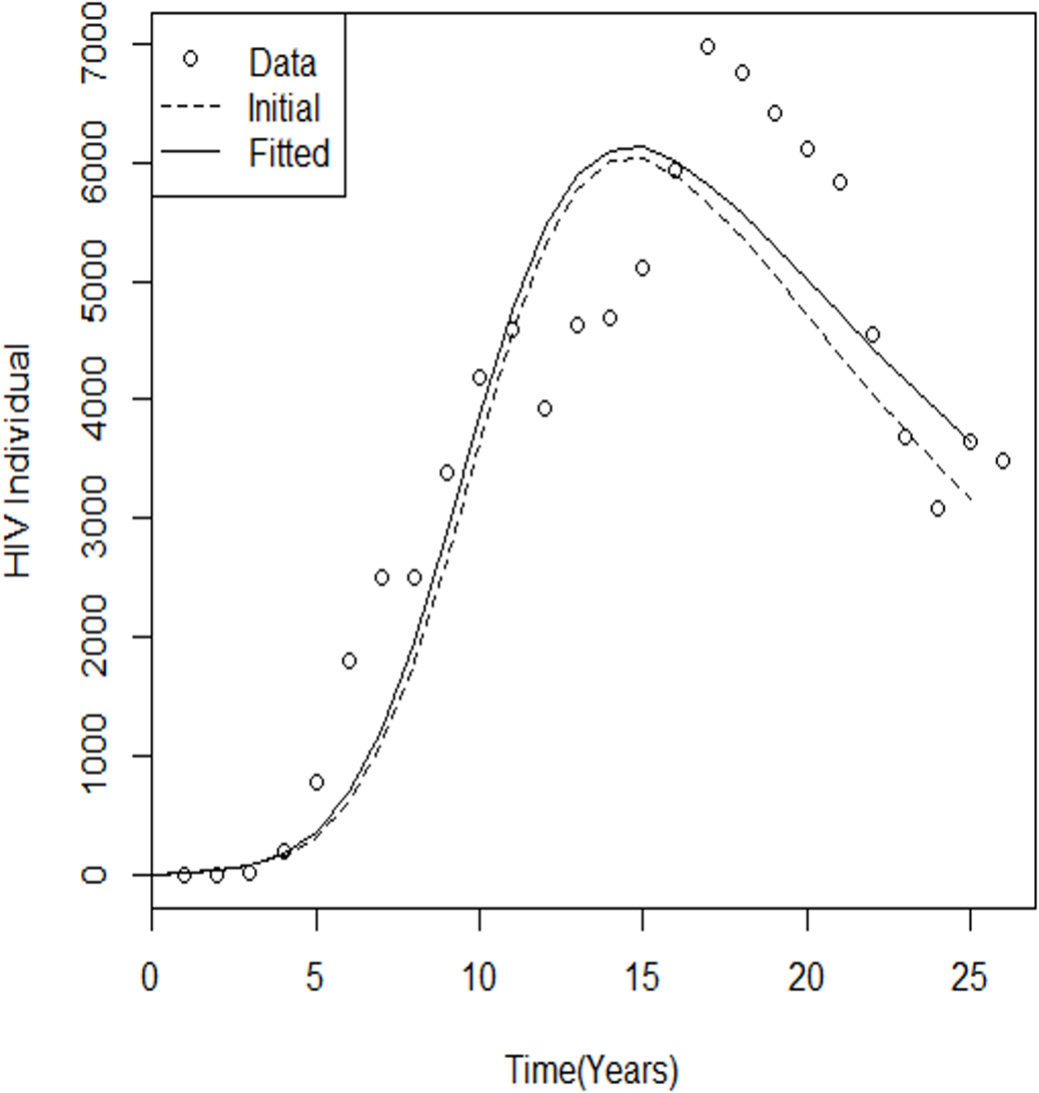
Best-fit and initial model run for HIV cases. Comparison of yearly reported HIV case simulations with fitted parameters during the 25-year (1986–2011) calibration and validation periods. Comparing and evaluating the performances of the plotted graph can be used for further studies. The dotted line represents the estimated parameters, with black representing the initial parameters.

**Fig 3:**
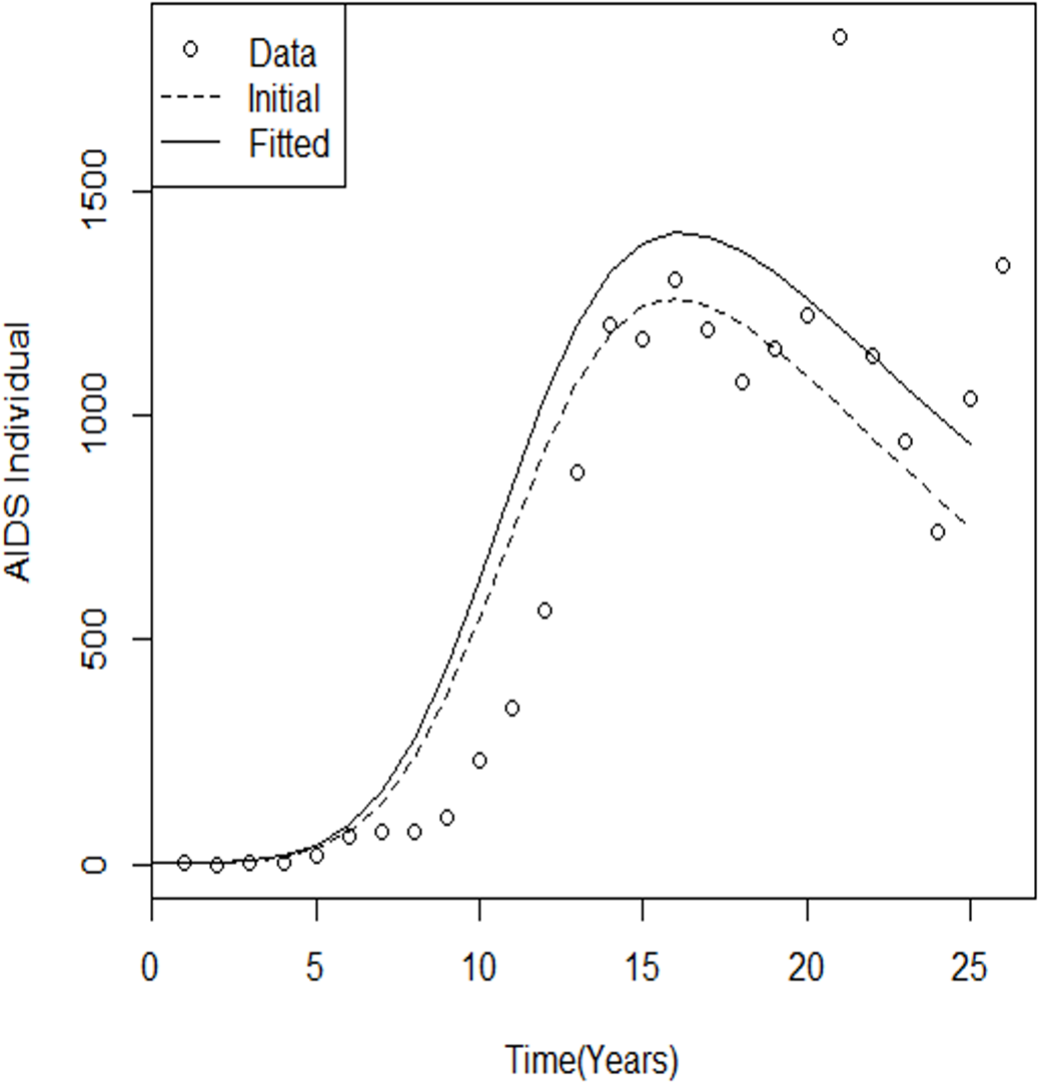
Best-fit and initial model run for AIDS cases. Comparison of yearly reported AIDS case simulations with fitted parameters during the 25-year (1986–2011) calibration and validation periods. Comparing and evaluating the performances of the plotted graph can be used for further studies. The dotted line represents the estimated parameters, with black representing the initial parameters.

For comparison, the initial model output and the best-fit model are plotted against the data shown in Fig 2 and Fig 3.

The following figures show the simulations from the MCMC approach for each parameter.

**Fig 4:**
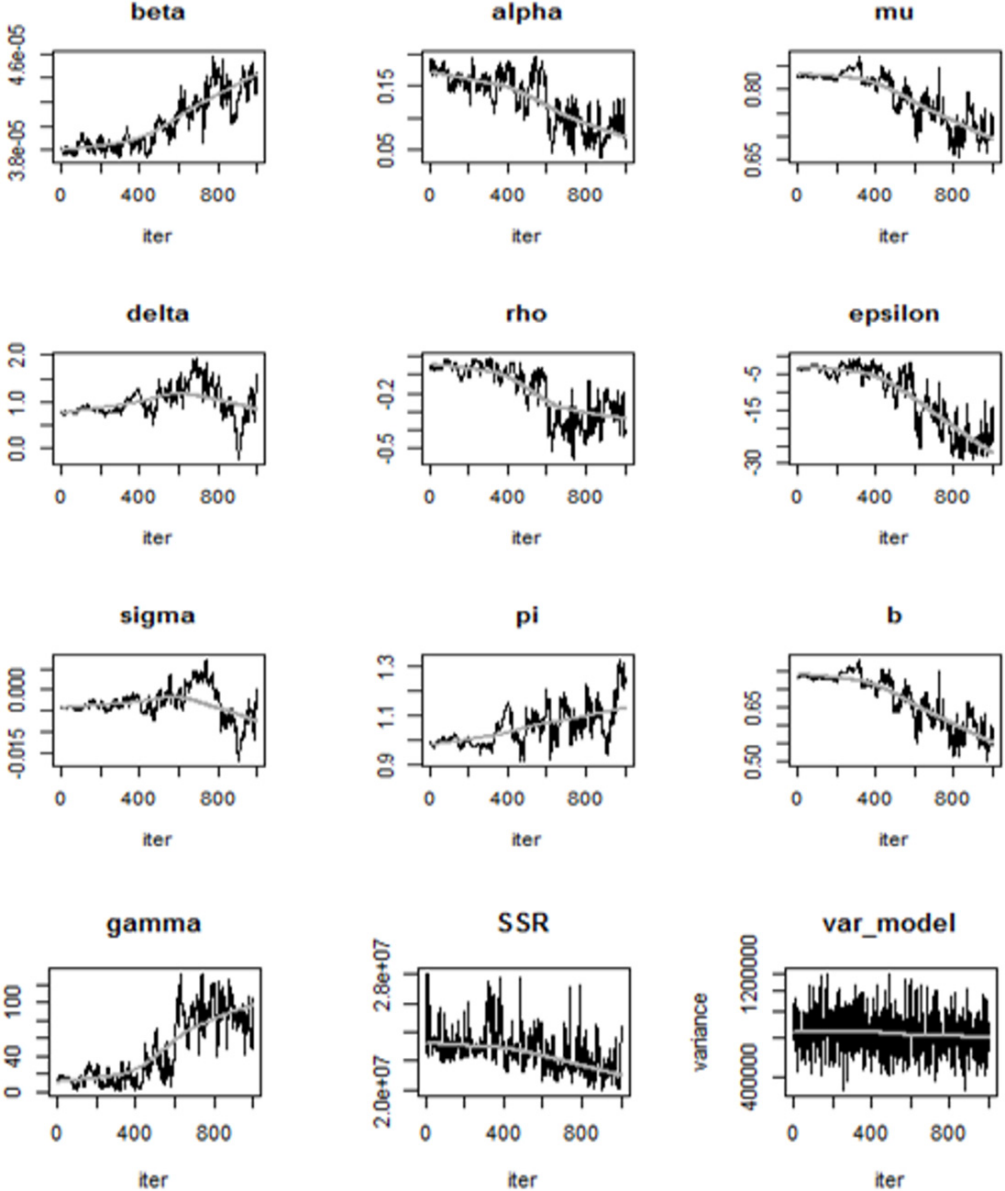
MCMC parameter values per iteration. The traces of the MCMC chain (grey line) show that the chain has converged, indicating that there is no apparent drift. The last Fig also shows the error variances for each observed variable.

From Fig 4, the traces of the MCMC chain (grey line) show that the chain has converged, which indicates that there is no apparent drift. The last figure also shows the error variances for each observed variable.

**Fig 5:**
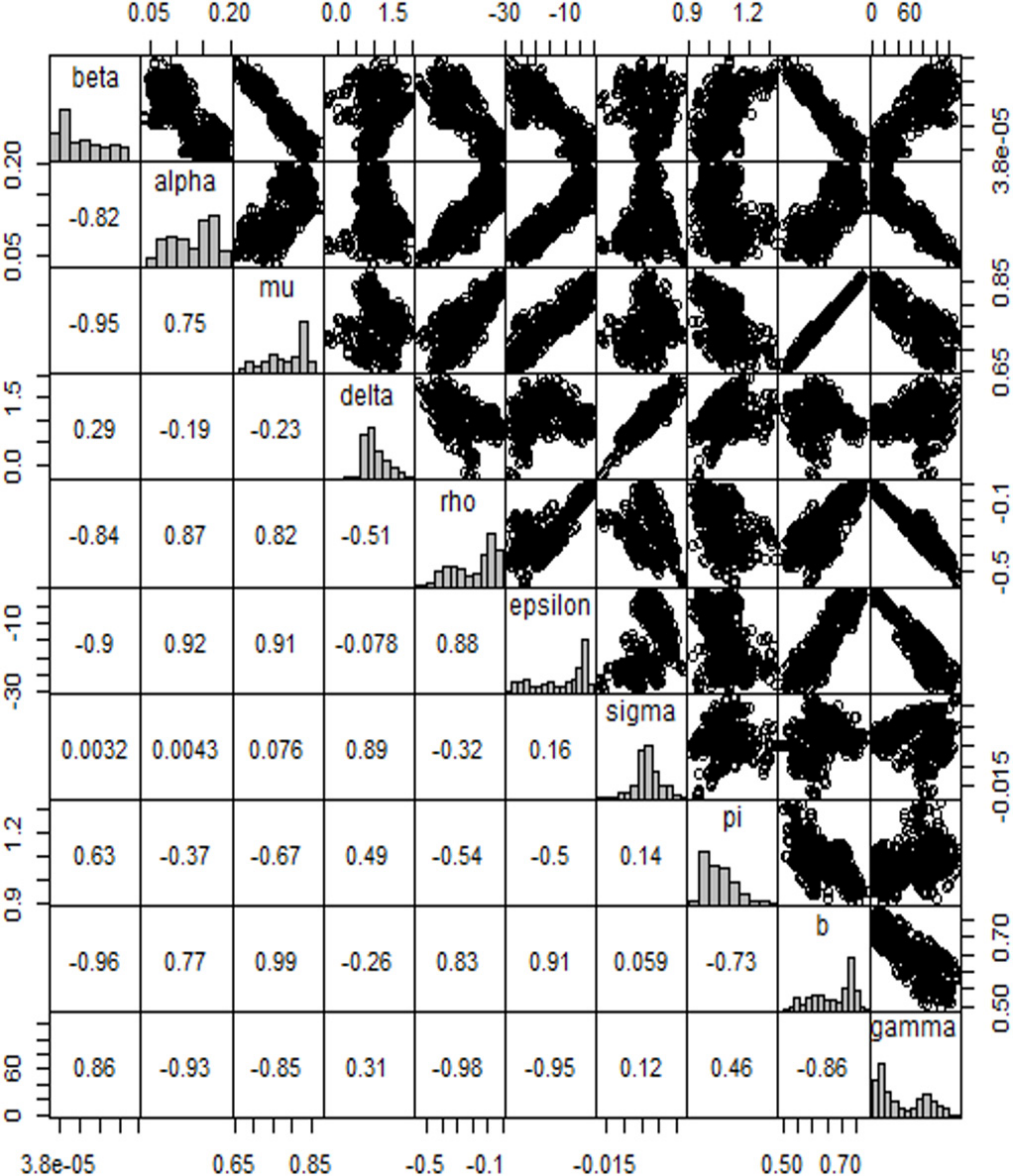
Pairs plot of the MCMC samples for the ten parameters. The pairs plot shows a strong relationship between parameters *γ* and *ρ* (the rate at which an individual will fully move from the *A*_1_(*t*) class to the *A*_2_(*t*) class and the disease-induced mortality rate for *A*_2_(*t*), respectively). This plot visualizes the pairwise relationship in the upper panel, the correlation coefficients in the lower panel, and the marginal distribution for each parameter, represented by a histogram, on the diagonal. This Fig also shows the correlation between mean HIV reported cases and the various parameters, as well as their positive relationships.

In Fig 5, the pairs plot shows a strong relationship between parameters *γ* and *ρ* (the rate at which an individual will fully move from *A*_1_(*t*) class to *A*_2_(*t*) class and the disease-induced mortality rate for *A*_2_(*t*), respectively). This plot visualizes the pairwise relationship in the upper panel, the correlation coefficients in the lower panel, and the marginal distribution for each parameter, represented by a histogram, on the diagonal. This figure also shows the correlation between the mean HIV reported cases and the various parameters, as well as their positive relationships.

**Fig 6:**
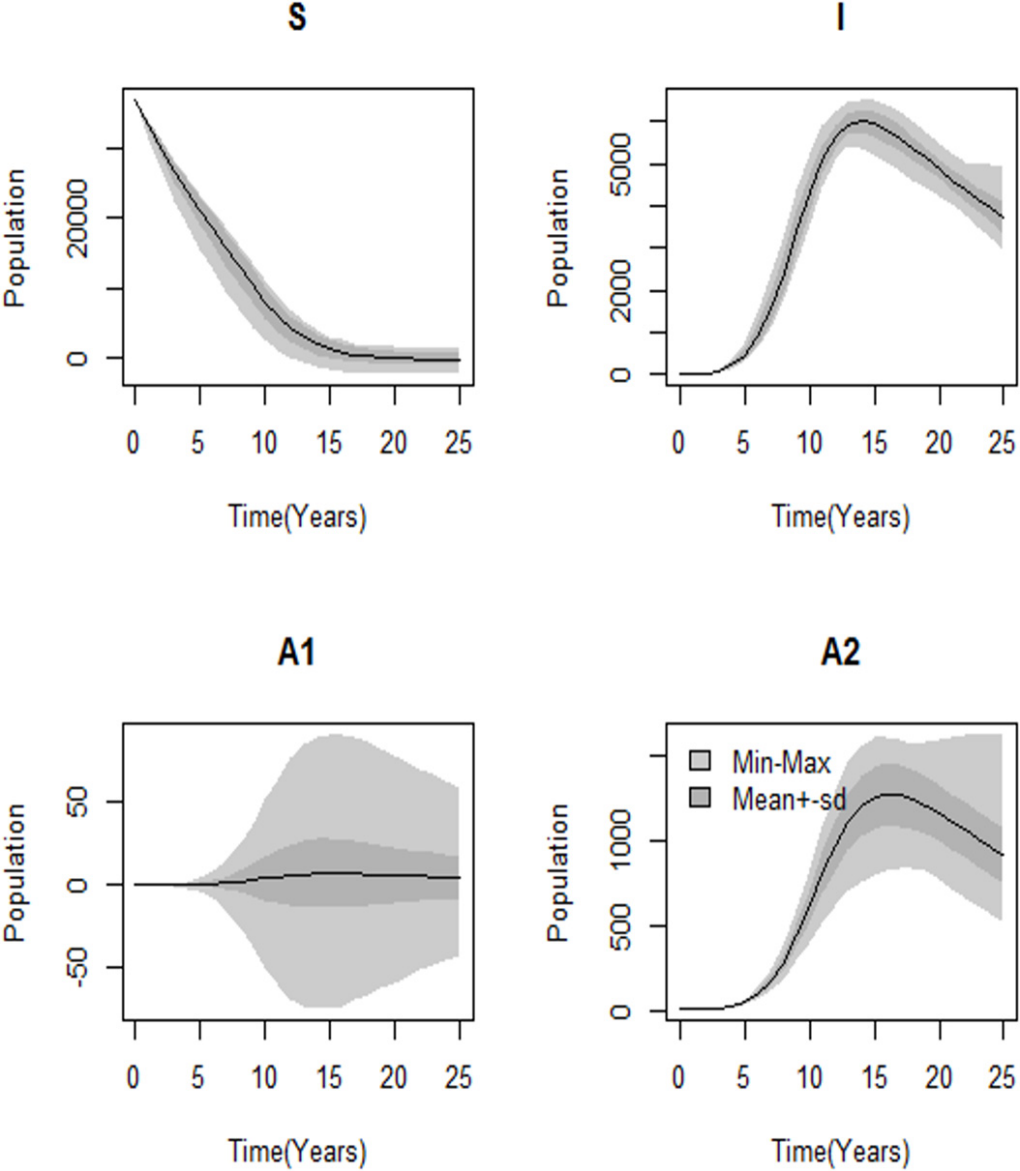
Sensitivity range of yearly reported HIV and AIDS cases. The high variances were observed in the following compartment order: *A*_1_ > *A*_2_ > *I* > *S*. This shows that there was predictive accuracy of the model reflected by the variance of the predictive distribution. The large number for the variance is due to either the uncertainties in the model or noise in data collection, and the model fit the noisy data reasonably well.

As shown in Fig 6, the high variances were observed in the following compartment order: *S* > *I* > *A*_2_ > *A*_1_, which shows the predictive accuracy of the model reflected by the variance of the predictive distribution. The large variance is due to either the uncertainties in the model or noise in data collection, and this model fit the noisy data reasonably well.

## Conclusion

This study demonstrates how to model the spread of AIDS after HIV infection. As with any modeling study of such a complex system as HIV and AIDS, several assumptions were necessary to make the analysis tractable. We assumed that there were sexual interactions between the susceptible and HIV-infected populations, that infected newborn babies moved directly to the HIV class and that a fraction of the remaining population also moved to the susceptible class to increase the growth of the total population. The model also assumed that *γ* is the rate at which an individual will fully move from *A*_1_(*t*) class to *A*_2_(*t*) class; this rate was not considered by the model reported in a previous study [14]. HIV/AIDS continues to infect the susceptible population if no control measures are swiftly enacted, and the endemic point, if it existed, could have been stable if all the eigenvalues were negative. The reproduction number *R*_0_ is a threshold value or number that determines the stability of the disease-free equilibrium. If *R*_0_ > 1, then an epidemic of AIDS occurs, and if *R*_0_ < 1, then the disease-free equilibrium is locally asymptotically stable and disease becomes endemic. Our results show that the disease-free steady state is unstable because the basic reproduction number *R*_0_ was 13.22657. These results show that the number of HIV cases and AIDS cases is still epidemic within the Malaysian population, and this will have policy implications for the most at-risk groups of populations, especially the HIV-infected population (Fig 2 and Fig 3). The public health implication of this instability is that HIV will continue to infect the susceptible population because in the rate of newborn babies *b*(*I* + *A*_1_ + *A*_2_), *b* is the parameter with the highest value compared with the other parameters. Thus, there must be effective intervention measures that will continue to minimize the spread of the HIV epidemic within the unaffected population. Furthermore, there must be effective ways to minimize the spread of pre-AIDS *A*_1_(*t*) cases that progress to AIDS after HIV infection, especially the rate of newborn babies *b*(*I* + *A*_1_ + *A*_2_), because this had the highest impact on disease spread and indicated that more infected HIV/AIDS individuals are born at these stages than at the other stages. Our results further suggest that without the intervention of antiretroviral medication (drug treatment), the rate *γ* at which an individual will fully move from the *A*_1_(*t*) class to the *A*_2_(*t*) class is 0.99/year. This information will assist policymakers in deciding at which stage to introduce intervention measures. The analysis presented herein with MCMC can be applied to a large class of HIV/AIDS epidemic models by taking into account both the uncertainty in the model parameters and other characteristics of the target posteriors by generating chains of samples. Contrasts were found as the posterior standard deviations exceeded the standard errors, as shown in Table 2 and Table 3. The graphical descriptions further demonstrate and support the empirical results and the long-term model prediction. We conclude that the predictive distributions generated predicted the model to a large degree of accuracy, as shown in Fig 6. Finally, there were some significant differences in the estimated parameters that will be useful to public health, potentially representing a practical and more effective way to epidemiologically model AIDS disease after HIV infection.

**Table 3:** Empirical mean, standard deviation for each variable and standard error of the mean.

	Mean	SD	Naive SE	Time-series SE
*β*	3.849e-05	1.016e-06	3.213e-08	2.270e-07
*α*	1.645e-01	2.920e-02	9.233e-04	5.265e-03
*μ*	8.637e-01	3.543e-02	1.120e-03	9.907e-03
*δ*	8.178e-01	1.419e-01	4.488e-03	5.016e-02
*ρ*	6.209e-02	3.264e-02	1.032e-03	9.058e-03
*ε*	1.037e-01	4.467e+00	1.413e-01	1.162e+00
*σ*	6.448e-03	2.675e-03	8.458e-05	4.851e-04
*π*	1.078e+00	8.876e-02	2.807e-03	2.821e-02
*b*	7.544e-01	2.938e-02	9.291e-04	9.073e-03
*γ*	1.076e+01	8.566e+00	2.709e-01	2.182e+00

Where *β* = the contact rate between susceptible individuals and exposed or HIV-infected individuals, *α* = removal rate, *μ* = nature death rate, *δ* = the portion of HIV-infected individuals, *ρ* = disease-induced mortality rate of *A*_1_(*t*), *ε* = disease-induced mortality rate of *A*_2_(*t*), *σ* = disease-induced mortality rate of *I*(*t*), *π* = the portion of individuals infected with HIV, *b* = birth rate, *γ* = is the rate at which an individual will fully move from *A*_1_(*t*) class to *A*_2_(*t*) class.

## References

[1] (July 21, 2014) World Health Organisation-HIV department, Global summary of the HIV/AIDS epidemic, December 2013.

[2] Hyman JM, Ann Stanley E (1989) Using mathematical models to understand the aids epidemic. Mathematical and Computer Modelling 12: 1180.

[3] Romieu I, Sandberg S, Mohar A, Awerbuch T (1991) Modeling the AIDS Epidemic in Mexico City. Human biology 63: 683–695 1916742

[4] Nyabadza F, Mukandavire Z, Hove-Musekwa SD (2011) Modelling the HIV/AIDS epidemic trends in South Africa: Insights from a simple mathematical model. Nonlinear Analysis: Real World Applications 12: 2091–2104.

[5] Naresh R, Tripathi A, Omar S (2006) Modelling the spread of AIDS epidemic with vertical transmission. Applied Mathematics and Computation 178: 262–272.

[6] Merli MG, Hertog S, Wang B, Li J (2006) Modelling the Spread of HIV/AIDS in China: The Role of Sexual Transmission. Population Studies 60: 1–22. 16464772 10.1080/00324720500436060

[7] Kakehashi M (1999) A mathematical analysis of the spread of HIV/AIDS in Japan (vol 15, pg 299, 1998). Ima Journal of Mathematics Applied in Medicine and Biology 16: 111–112. 9951712

[8] de Arazoza H, Lounes R (2002) A non‐linear model for a sexually transmitted disease with contact tracing. Mathematical Medicine and Biology 19: 221–234. 12650336

[9] Kim J-H (2009) Dynamic partnerships and HIV transmissions by stage [3392859]. United States—Michigan: University of Michigan.

[10] Haario H, Laine M, Mira A, Saksman E (2006) DRAM: Efficient adaptive MCMC. Statistics and Computing 16: 339–354.

[11] Petzoldt T, Soetaert K (2010) Inverse Modelling, Sensitivity and Monte Carlo Analysis in R Using Package FME. Journal of Statistical Software 33.

[12] Slimi R, El Yacoubi S, Dumonteil E, Gourbière S (2009) A cellular automata model for Chagas disease. Applied Mathematical Modelling 33: 1072–1085.

[13] Apenteng OO (2009) Demographic Modelling of Human Population Growth.

[14] Rao ASRS (2003) Mathematical modelling of AIDS epidemic in India. 84 1192–1197.

[15] Brun R, Reichert P, Künsch HR (2001) Practical identifiability analysis of large environmental simulation models. Water Resources Research 37: 1015–1030.

[16] Laine M (2008) Adaptive MCMC Methods with Applications in Environmental and Geophysical Models: Finnish Meteorological Institute.

[17] Soetaert K, Herman PMJ (2009) A Practical Guide to Ecological Modelling: Using R as a Simulation Platform: Springer.

[18] Gelman A, Carlin JB, Stern HS, Dunson DB, Vehtari A, Rubin DB (2013) Bayesian Data Analysis; Hall/CRC Ca, editor. Boca Raton.

[19] van den Driessche P, Watmough J (2005) Reproduction numbers and sub-threshold endemic equilibria for compartmental models of disease transmission. Mathematical Biosciences 180: 29–48. 10.1016/s0025-5564(02)00108-6 12387915

[20] Jones JH (2007) Notes On R0. Califonia: Department of Anthropological Sciences. 17 p.

[21] Silva CJ, Torres DFM (2013) Optimal control for a tuberculosis model with reinfection and post-exposure interventions. Mathematical Biosciences 244: 154–164. doi: 10.1016/j.mbs.2013.05.005 23707607 10.1016/j.mbs.2013.05.005

[22] Diekmann O, Heesterbeek JAP, Roberts MG (2010) The construction of next-generation matrices for compartmental epidemic models. Journal of the Royal Society Interface 7: 873–885. 10.1098/rsif.2009.0386 PMC2871801 19892718

[23] Roberts M, Heesterbeek J (2007) Model-consistent estimation of the basic reproduction number from the incidence of an emerging infection. Journal of Mathematical Biology 55: 803–816. 17684743 10.1007/s00285-007-0112-8 PMC2782110

[24] (2012) Ministry of Health, Malaysia 2012 Global AIDS response country progress report.

